# Neural and behavioral signatures of social evaluation and adaptation in childhood and adolescence: The Leiden consortium on individual development (L-CID)

**DOI:** 10.1016/j.dcn.2020.100805

**Published:** 2020-07-11

**Authors:** Eveline A. Crone, Michelle Achterberg, Simone Dobbelaar, Saskia Euser, Bianca van den Bulk, Mara van der Meulen, Lina van Drunen, Lara M. Wierenga, Marian J. Bakermans-Kranenburg, Marinus H. van IJzendoorn

**Affiliations:** a Institute of Psychology, Leiden University, The Netherlands; b Leiden Institute for Brain and Cognition, Leiden University, The Netherlands; c Department of Clinical Child and Family Studies, VU Amsterdam, The Netherlands; d Department of Psychology, Education and Child Studies, Erasmus University, The Netherlands; e School of Clinical Medicine, University of Cambridge, United Kingdom

**Keywords:** Cognitive control, Self regulation, Prosocial, Differential susceptibility, Brain development

## Abstract

The transition period between early childhood and late adolescence is characterized by pronounced changes in social competence, or the capacity for flexible social adaptation. Here, we propose that two processes, self-control and prosociality, are crucial for social adaptation following social evaluation. We present a neurobehavioral model showing commonalities in neural responses to experiences of social acceptance and rejection, and multiple pathways for responding to social context. The Leiden Consortium on Individual Development (L-CID) provides a comprehensive approach towards understanding the longitudinal developmental pathways of, and social enrichment effects on, social competence, taking into account potential differential effects of such enrichment. Using Neurosynth based brain maps we point towards the medial prefrontal cortex as an important region integrating social cognition, self-referential processing and self-control for learning to respond flexibly to changing social contexts. Based on their role in social evaluation processing, we suggest to examine medial prefrontal cortex connections with lateral prefrontal cortex and the ventral striatum as potential neural differential susceptibility markers, in addition to previously established markers of differential susceptibility.

## 1 Social competence and neural development

Social competence, which is defined as the cognitive, emotional and social skills that are needed for social adaptation (Denham et al., 2003; Huber et al., 2019; Rydell et al., 1997), is one of the most important requirements for developing social relations. Social competence is particularly needed when dealing with social evaluations, providing us with signals of acceptance and rejection, which foster feelings of positive or negative self-evaluation (Yoon et al., 2018). In case individuals are personally confronted with rejection, this may trigger socially adaptive self-protection, such as reassessing the value of the messenger (Overall and Sibley, 2009), or displaying aggression following rejection (Chester et al., 2018). In contrast, rejection that is directed towards others (i.e., observed social evaluation) may trigger other-oriented prosocial behaviors, such as helping and comforting (Masten et al., 2011; Rydell et al., 1997). Thus, social competence entails important mechanisms involved in the adaptive responses associated with rejection and acceptance of self and others. This review is organized around the common themes of how positive and negative social evaluations can trigger self-protective and other-oriented processes, and how individuals may differ in their susceptibility to these social experiences.

**Fig. 1:**
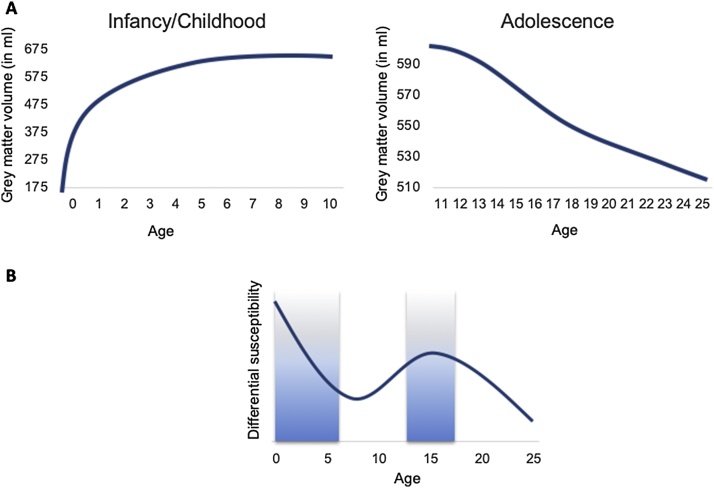
(A) Windows of larger cortical reorganization in infancy/early childhood (0-10 years; based on Gilmore et al., 2018) and during adolescence (10-25-years; based on Tamnes et al., 2017). (B) Potential age-related windows of larger effects of differential susceptibility on social competence increasing the developmental differences between children with lower versus higher susceptibility.

Growing evidence suggests that the cognitive, emotional and social processes that are important for adaptive social behavior show continued developmental changes during childhood and adolescence. Whereas adults have developed (self-) adaptative mechanisms in response to experienced or observed negative social feedback, these mechanisms may not yet be in place during childhood and adolescence, making this a period of higher sensitivity to social evaluation signals (Rodman et al., 2017). The study of these mechanisms has benefited from recent insights from neuroscience research, which pointed to prolonged windows of grey matter increases and reductions. These grey matter changes possibly mark periods of greater sensitivity to environmental influences. Particularly the period of infancy and toddlerhood (0–4 years) has been suggested to be marked by rapid neuronal growth and cortical growth and pruning (Girault et al., 2019), which stabilizes after the age of approximately 4 years (Gilmore et al., 2018). This pattern is followed by a second window of cortical reorganization in terms of a rapid reduction of gray matter in puberty, with a less steep decrease and stabilization in late adolescence until the early twenties (Tamnes et al., 2017) (see Fig. 1A). We propose that these periods in development provide windows of increased plasticity and refinement of neural development and a relative larger susceptibility to environmental influences (Fig. 1B), such as supporting or aversive experiences with parents and peers.

Insights in the effects of social evaluation on neural processes at a micro level (i.e., instant social experiences) have been informed by the use of functional magnetic resonance neuroimaging (fMRI) paradigms signaling acceptance and rejection by others. Functionally, prior studies suggest a coordinating role of the medial prefrontal cortex when responding to social evaluation (Yoon et al., 2018). The medial prefrontal cortex is generally considered a hub region for different processes that are related to self-other referential processing. It has been considered part of the brain network involved in social cognition, together with the temporal parietal junction (TPJ), superior temporal cortex (STS) and anterior temporal lobe (Blakemore and Mills, 2014). The medial prefrontal cortex is also part of the self-referential cortical midline network together with the posterior parietal cortex/precuneus (Pfeifer and Peake, 2012). Finally, medial prefrontal cortex has been implicated as part of the affective-control network, together with the ventral striatum (van Duijvenvoorde et al., 2016). Therefore, many studies have now pointed towards the medial prefrontal cortex as an important region for adaptive responses to social evaluation (Yoon et al., 2018). Here, we review the processes that are important for social competence development in the context of social evaluation of self (experienced social evaluation) and others (observed social evaluation), based on neural and behavioral evidence. Regulating emotions in response to social evaluations of self is expected to be associated with behavioral profiles of self-control, whereas responding to needs of others when observing social evaluations is expected to be associated with behavioral profiles of prosociality (see Table 1 for constructs and definitions).

**Table 1:** Definitions of constructs.

**Social competence**: the cognitive, emotional and social skills needed for social adaptation
**Social evaluation**: evaluations by self and by others that may or may not trigger the need for
adaptation
**Social adaptation**: flexible adjustment to changing social environments
**Self-control**: regulating emotions and behaviours in response to social evaluation of self and others
**Self-protection**: protecting favourable self-views following negative social evaluation
**Self-esteem**: the feeling of self-worth
**Prosocial behaviour**: any action that serves to benefit another person

The study of neural responses to experimental tasks that mimic social evaluation experiences may provide a powerful means to understanding the association between experimentally induced moment-to-moment experiences of social evaluation and more prolonged real-life social experiences that may affect the development of social competence processes. Many scientists have examined prolonged developmental trajectories of social competence, possibly because in periods of developmental change the processes involved can be fostered and shaped through social experiences (Blakemore and Mills, 2014; Dahl et al., 2018). The social environment of a child changes considerably between birth and young adulthood, and this may provide unique risks and opportunities for shaping and fostering processes that are important for responding to social evaluation. Whereas children from birth to early childhood (ages 0–7 yrs) are most strongly influenced by their parents and siblings, this context gradually extends to include a stronger influence of peers in middle childhood and adolescence (8–17 yrs). This transition coincides with the onset of puberty, which is the period in life characterized by a transition to extending social relations outside the family context (Crone and Dahl, 2012). The development of many aspects of social competence eventually leads to the development of young adults with social goals that they find worthwhile or are required to pursue (18–25 yrs), and adaptive mechanisms for responding to self and other social evaluation.

An important question concerns how various social environments hamper or foster the development of social competence. Empirical evidence based on randomized-control intervention studies suggest that, in addition to possible sensitive windows within development, not all individuals are similarly susceptible to influences of the environment on future outcomes (Belsky and de Haan, 2011; Belsky and van IJzendoorn, 2017). The differential susceptibility theory proposes that some children are more susceptible than their peers to their positive or negative social and physical environment, for better *and* for worse, such that their social competence is fostered to a greater extent in a positive environment, but also harmed more in a negative environment compared to individuals who are less susceptible to environmental influences. An experimental ‘micro-trial’ with Cyberball has shown, for example, that aggression in response to exclusion is differentially affected by (MAOA) genotype (Gallardo-Pujol et al., 2013), although this study was limited to the effects of a negative social environment (ostracism). Other experiments supported genetic differential susceptibility to both positive (i.e., environmental enrichment) and negative (i.e., deprivation) social environments (Bakermans-Kranenburg and Van IJzendoorn, 2015; Belsky and van IJzendoorn, 2017; Knop et al., 2020).

Within-person variation in susceptibility to social context has been proposed for neural development, where it is assumed that brain regions supporting cognitive, affective and social development develop over many years and are particularly influenced by adverse or enriched social environments (Schriber and Guyer, 2015; Tottenham, 2019). The developmental periods that are characterized by increased plasticity and refinement of neural development (early childhood, emerging adolescence) may however not only show a generally enhanced susceptibility to environmental influences, but may also be characterized by larger inter-individual differences in susceptibility (differential susceptibility, see Fig. 1B). However, it remains to be determined whether and how variations in parenting and other aspects of the social environment modulate the impact of social evaluation in different phases of development (sensitive windows) moderated by individual characteristics (differential susceptibility), and how these periods of accelerated neurobiological development and concomitant enhanced sensitivity increase the moderating influence of differential susceptibility markers, specifically the medial prefrontal cortex and its connections.

**Fig. 2:**
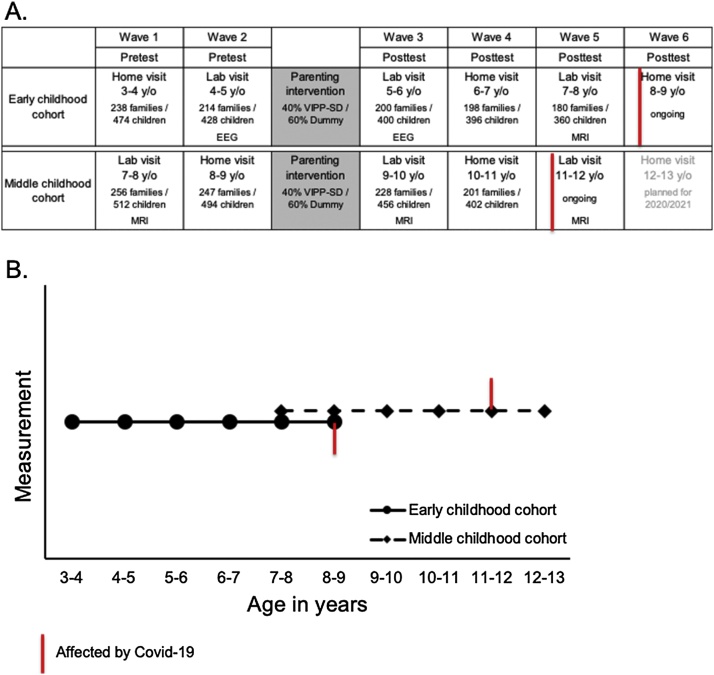
(A) LCID cohort design with 6 time points and overlapping waves. (B) Visualization of LCID cohort design with 6 time points and overlapping waves for studying social adaptation. Red marks indicate influence of the Covid-19 pandemic, which caused delays in assessments and required adaptation of home-based assessments to remote video assessments. (For interpretation of the references to colour in this figure legend, the reader is referred to the web version of this article).

In this review, we differentiate between social competence processes involved in social evaluation of self and associated self-control, and processes involved in witnessing the social evaluation of others and associated prosociality. Ideally, research should cover the whole developmental period from birth to adulthood. This review provides only the starting point of this approach with a relatively strong emphasis on childhood and early adolescence. The review is selective and covers behavioral and neurobiological measures that contribute to an integrative neurobehavioral perspective, and it is based on the latest and most robust findings. The results are organized around the common themes of the Leiden Consortium on Individual Development (L-CID); an experimental accelerated cohort-sequential longitudinal twin intervention-study that includes children between ages of 3−13-years (see Fig. 2 for the cohort approach and Appendix 1–3 for included measures, documentation and demographics) (Euser et al., 2016). The reason for starting at the age of 3–4 years is because this period can still be considered the tail of elevated differential susceptibility (Fig. 1B) while at the same time allowing for the use of experimental paradigms that are comparable across the whole developmental period from childhood to adulthood. The program is organized in two cohorts using an accelerated cohort-sequential longitudinal design, with starting ages at 3–4-years and 7−8-years to capture a 10-year developmental period in a 6-year-study. The ambition of the program is to extend measurements until early adulthood to include assessments in the second period of heightened differential susceptibility.

**Fig. 3:**
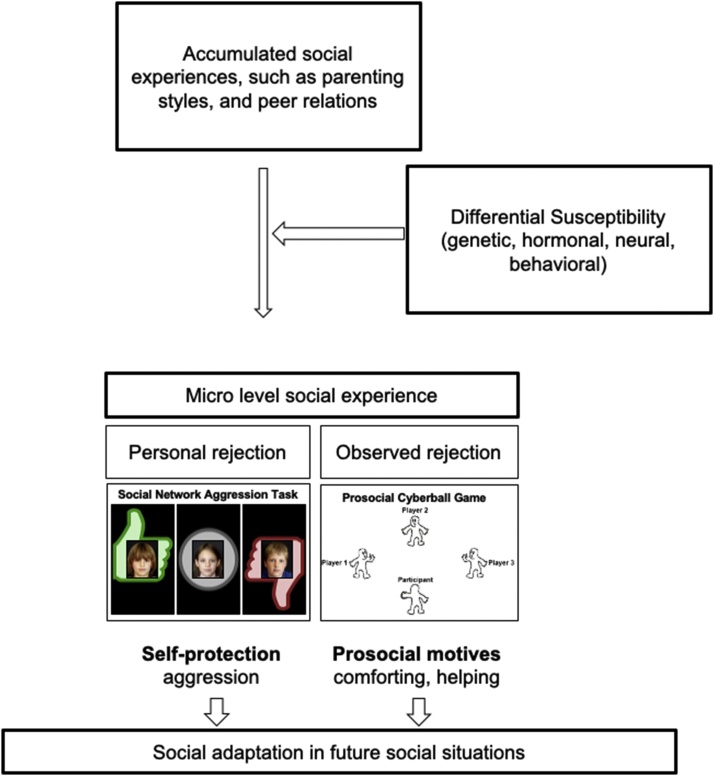
Conceptual model of the Leiden Consortium on Individual Development (L-CID) study. Social competence is examined in the context of social evaluation of self and other, for which experimental paradigms were developed. These social competence measures, as reflected in neural and behavioral responses, are expected to indicate social adaptation. This Figure focuses on experienced and observed social rejection, but similar processes may be in place for experienced and observed social acceptance.

The L-CID program has two aims: 1) to unravel the developmental trajectories, differences and commonalities of behavioral profiles and neural correlates for self and other-oriented social evaluation, which are two important components of social competence (see Fig. 3 for paradigm examples), and 2) to understand differential susceptibility to environmental enrichment (in a randomized control trial) in different phases of development, with a specific focus on early childhood and emerging adolescence. This review is organized along these lines.

## 2 Developmental trajectories of processing social evaluation

### 2.1 Neural development of self-control processes

Self-control is defined as regulating emotions and behaviors in response to social evaluation of self. Many prior studies have examined self-control in terms of behavioral control, such as withholding responses to no-go stimuli (Rubia et al., 2013) or delay of gratification (Christakou et al., 2011). Self-control is also of paramount importance in the context of social evaluation. Specifically, social evaluation affects self-esteem and, in case of rejection, harms the need for belonging (Williams and Nida, 2011). Strategic motives for self-protection may include down-evaluation of others (Overall and Sibley, 2009), temporal distancing (Ahmed et al., 2018) or aggression towards the source of rejection (Reijntjes et al., 2013). Prior research showed that children respond to the immediate social evaluation feedback in their responses towards others, whereas adults use more accumulated experiences in their response to others, taking the larger context into account (Yoon et al., 2018).

Several neuroimaging studies have examined the experience of social evaluation in adolescents and adults, by examining the neural responses to acceptance and rejection in a social feedback paradigm, in which participants receive feedback from other participants signaling whether they were liked (acceptance) or disliked (rejection) based on first impression (Gunther Moor et al., 2010a; Somerville et al., 2006). These studies showed that in adolescents and adults, the experience of acceptance led to the activation of a wide network of brain regions including the amygdala and the ventral striatum, whereas rejection was associated with stronger social salience detection including activity in the insula and prefrontal cortex, especially for older adolescents (Gunther Moor et al., 2010b; Guyer et al., 2012).

To examine self-control in relation to social evaluation, Achterberg and colleagues developed a novel neuroimaging paradigm, which is referred to as the Social Network Aggression Task (SNAT) appropriate for older children, adolescent and adults (Achterberg et al., 2016a). This paradigm is based on studies in adults in which it was reported that experiencing rejection in a ball tossing game was associated with more aggression in the form of blasting a loud noise (Chester et al., 2018). It was found that indeed adults and children respond with larger noise blast aggression to rejection feedback compared to acceptance feedback (Achterberg et al., 2016b, 2017). Even 4−6-year-old children popped more balloons of another player in a child-appropriate version of the Social Network Aggression Task- Early Childhood (SNAT-EC) (van Wijk et al., 2019b).

This paradigm was developed to also examine neural responses to acceptance and rejection feedback using fMRI. By including a neutral condition to the SNAT-paradigm, it was possible to examine the brain regions that were generally sensitive to social feedback (positive or negative) and regions that were responding to the specific valence of feedback (negative > positive). Prior research had left undecided which brain regions were responding to valence (Guyer et al., 2012; Somerville et al., 2006), saliency (Vijayakumar et al., 2017) or incongruence with prior expectations (Somerville et al., 2006). A study in 18−25-year old adults using the SNAT revealed that experiencing both acceptance (positive) and rejection (negative), relative to neutral feedback, resulted in strong activation in bilateral insula and medial prefrontal cortex (Achterberg et al., 2016b). This activation was interpreted as signaling social saliency, given that acceptance and rejection feedback may trigger the need for action more than the neutral condition. Consistent with studies on reward processing (Liu et al., 2011), social acceptance relative to rejection was associated with stronger activity in the ventral striatum, suggesting that social acceptance and reward processing rely on the same brain regions (Liu et al., 2011). Social acceptance relative to rejection also elicited strong activation in supplementary motor area and bilateral DLPFC, regions previously associated with control processes, possibly indicating that acceptance is associated with approach processes (Tyborowska et al., 2016). In these young adults, rejection relative to acceptance feedback specifically resulted in stronger activation in medial prefrontal cortex, in a region more anterior/superior of the medial prefrontal cortex for social salience, a region often implicated in social cognition (Burnett et al., 2011).

**Fig. 4:**
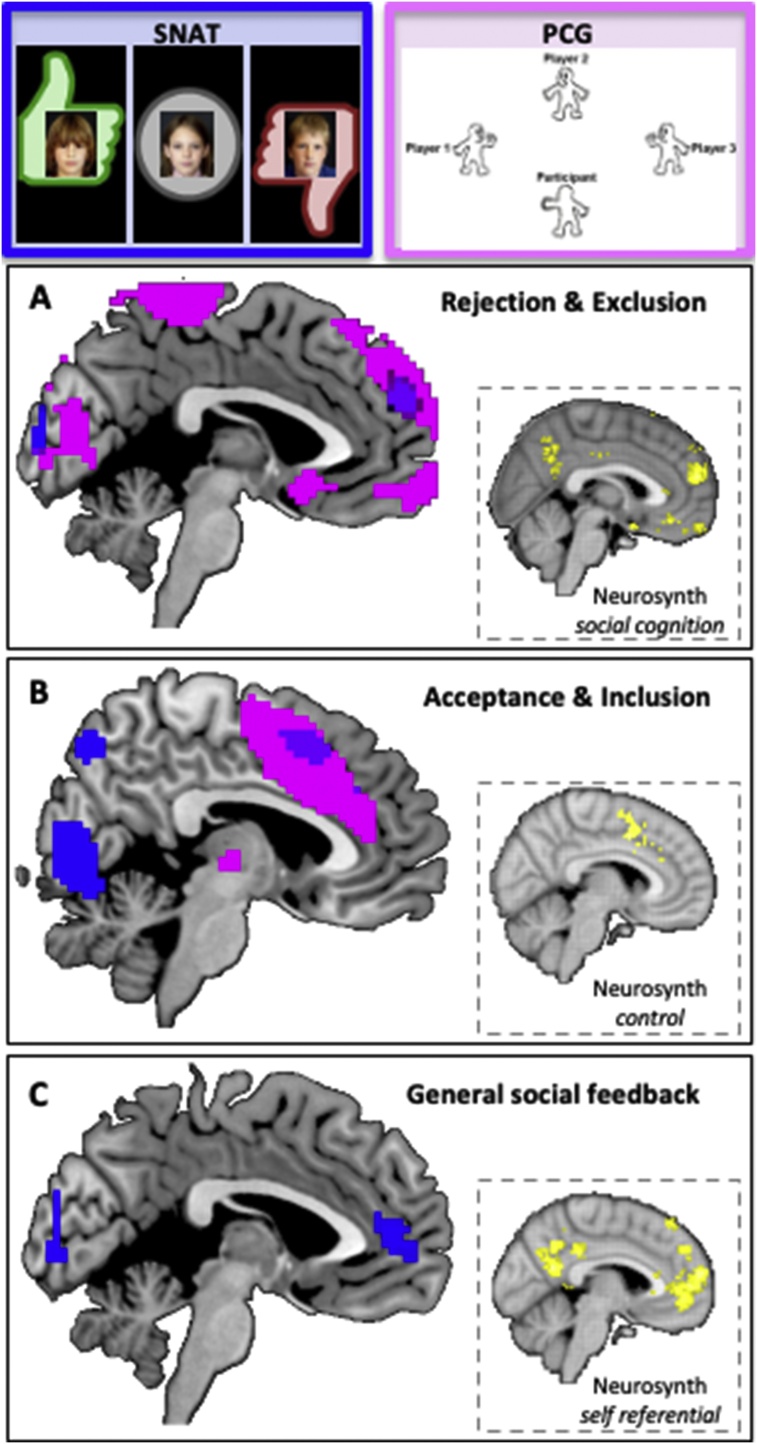
Overlapping neural activation in two social evaluation paradigms for **(A)** rejection and exclusion (superior medial PFC), **(B)** acceptance and inclusion (dorsal medial PFC), **(C)** social evaluation, and self-referential processing for social evaluation in general (acceptance & rejection vs neutral). The results are presented in Achterberg et al. (2018) and Van der Meulen et al. (2018) reporting on the same 7-9-year old participants of wave 1 of the middle childhood cohort (see Fig. 2, Fig. 3).

The same paradigm was also used separately in 7−10-year old children (Achterberg et al., 2017) and 7–9-year-old twins as part of the L-CID project (Achterberg et al., 2018a). These studies replicated the social saliency effect of acceptance/rejection feedback relative to neutral feedback in the bilateral insula and medial prefrontal cortex, and the increased activation in superior medial prefrontal cortex following rejection (see Fig. 4 for activation patterns in the medial prefrontal cortex).

In the SNAT paradigm participants were given the opportunity to retaliate following negative feedback, compared to positive and neutral feedback, by blasting a noise towards the source of the social feedback. Interestingly, stronger activation in DLPFC following rejection was associated with shorter noise blasts, that is, less aggression following rejection, in both adults (Achterberg et al., 2016b) and 7–9-year-old children (Achterberg et al., 2018b). A longitudinal comparison across the ages 7–9 to 9−11-years confirmed that those children who showed more DLPFC activation following rejection across time, also showed less aggression following rejection across time (Achterberg et al., 2020). Thus, whereas rejection-related activity was observed in medial prefrontal cortex, lateral prefrontal cortex differentiated more between behavioral responses to rejection and acceptance feedback.

Taken together, these studies suggest that social evaluation and associated self-control rely on both medial and lateral prefrontal cortex, together with the bilateral insula and ventral striatum. The experience of social evaluation was associated with activation in medial prefrontal cortex, consistent with prior studies showing that distinct subregions in the medial prefrontal cortex are involved in processing social evaluative cues (Yoon et al., 2018). The control of aggression following negative feedback was more strongly associated with activation in lateral prefrontal cortex, possibly indicating self-control of aggressive behavior (Casey, 2015). Three additional questions need to be investigated in more detail in future research. First, it is not yet known whether some individuals are less responsive to the social rejection feedback in general, with consequently less need to engage in self-control strategies. Less social responsiveness has been found to lower the impact of a prosocial peer on prosocial behavior of eight-years old children (Wildeboer et al., 2017). In future studies, we aim to unravel whether the same might be true for the impact of social rejection. Second, the network of brain regions that is involved in the SNAT paradigm has previously been associated with both emotion generation (including the amygdala, insula and medial PFC) as well as emotion regulation (including medial PFC and DLPFC) (Silvers and Guassi Moreira, 2019). Especially for SNAT-induced emotion regulation, an interesting question concerns whether individuals employ the capacity to self-regulate emotions using specific cognitive strategies (previously examined using cognitive reappraisal paradigms (Silvers et al., 2016)) versus the internally driven tendency to self-regulate emotions, which may be more sensitive to contextual factors (Silvers and Guassi Moreira, 2019). Future studies may test these questions using more detailed individual indices of general tendencies to self-regulate in daily life. Third, one prior transcranial direct current stimulation study in adults showed that lateral prefrontal cortex stimulation was directly related to a reduction of aggression following social exclusion (Riva et al., 2015). It is currently not known whether DLFPC is causally related to subsequent reduction of aggression in childhood studies. However, the longitudinal brain change- behavioral change relations suggest that DLPFC and aggression control are meaningfully related (Achterberg et al., 2020).

Even though few studies have examined social evaluation processes across development, developmental trajectories have been observed for the ability to control behavior in a non-social context, such as for the ability to delay gratification. When deciding between smaller immediate rewards or larger future rewards, impulsivity declines between childhood and adulthood (Peper et al., 2018). It currently remains to be determined whether self-control relies on common mechanisms in non-social versus social contexts. Delay of gratification is associated with stronger connectivity between the ventral medial prefrontal cortex (VMPFC), the dorsal lateral prefrontal cortex (DLPFC) and the ventral striatum (VS) (Achterberg et al., 2016a; van den Bos et al., 2015). Several studies revealed that the ability to delay gratification matures across childhood and adolescence, and adolescent decision-making is characterized by stronger VMPFC-VS connectivity compared to adulthood (Christakou et al., 2011), whereas delay-decisions in adults are characterized by stronger VMPFC-DLPFC connectivity compared to adolescents (van den Bos et al., 2015). Possibly, behavioral control in both social and non-social situations relies on a larger network of regions within the lateral and medial prefrontal cortex. Social evaluation and impulsive responses may rely in particular on medial prefrontal cortex regions, whereas delaying gratification and controlling anger may rely more on lateral prefrontal cortex (Crone and Steinbeis, 2017).

### 2.2 Neural development of prosociality

The experience of social inclusion and exclusion can be directed to self, but can also be observed in others. Witnessing social exclusion of others can lead to punishment of excluders (Will et al., 2013) and to prosocial behavior towards victims, such as helping and comforting (Masten et al., 2011). Prosocial behavior, which is defined as behavior that is directed to others without direct benefit to self, is particularly important to build and maintain social relationships over time. Recent studies have suggested that prosocial behavior is strongly dependent on context (van IJzendoorn and Bakermans-Kranenburg, 2014), such as whether participants are interacting with known (parents, peers) or unknown others (strangers) and whether participants know that they are being observed (Van Hoorn et al., 2016). The differentiation between prosocial actions towards known versus unknown others start in early childhood and emerges rapidly around middle childhood (between ages 9 and 12 years) (Guroglu et al., 2014), making the period of middle childhood and early adolescence particularly important for further distinguishing between ingroup-outgroup relations and caring about reputation (Hillebrandt et al., 2011).

A particular strong paradigm to trigger feelings of social exclusion is the Cyberball paradigm (Williams and Jarvis, 2006). This computerized three-player ball-tossing game involves unexpected exclusion of the participant by two other participants. Many prior studies revealed that the experience of social exclusion elicits strong feelings of negative affect and loss of control (Eisenberger et al., 2003), an experience that is heightened in mid adolescence (Sebastian et al., 2010). Meta-analyses have confirmed that the orbitofrontal cortex, together with the insula, is active when participants experience social exclusion (Cacioppo et al., 2013), as well as the anterior cingulate cortex in participants who have a history of experienced social exclusion (DeWall et al., 2012; van Harmelen et al., 2014). Interestingly, studies have shown that observed exclusion leads to increased neural activation in medial prefrontal cortex, especially in participants who report higher levels of empathy (Masten et al., 2011).

Prior studies that made use of two-person interaction games that include a division of goods (also referred as economic games) reported that social distribution behavior benefitting self and others is associated with a network or regions including the medial prefrontal cortex, superior temporal cortex and temporal-parietal junction (TPJ) (Rilling and Sanfey, 2011). Participants who experienced exclusion in a Cyberball game subsequently punished excluders in an economic game, by giving them fewer goods. This punishing behavior was associated with increased activation in insula and pre-supplementary motor area. In contrast, refraining from punishment (i.e., fair division of goods after being socially excluded) was associated with increased activation in social brain regions such as the medial prefrontal cortex and TPJ, and correlated with self-reported perspective taking (Will et al., 2015a, 2015b).

These studies inspired the study of helping behavior in the context of social evaluation using an adapted prosocial Cyberball Game (PCG) (Riem et al., 2013). This game consists of four players, one being the participant, who toss balls to each other. Two players exclude the third player, and the participant has the opportunity to help the excluded player by tossing more balls to this player (see Fig. 3). A prior study showed that such helping behavior is observed over the course of childhood and adolescence (ages 9−17-years) (Vrijhof et al., 2016). This task was administered in the L-CID study, in which it was observed that 7–9-year-old children show similar levels of helping as adults, although there was substantial variation between participants (van der Meulen et al., 2018). Together, these behavioral studies suggest that prosocial helping is a developmentally early emerging type of prosocial behavior.

Brain imaging studies using the PCG revealed that in adults, tossing to the excluded player is associated with more activation in the ventral striatum and the TPJ, regions that are often implicated in reward processing and perspective taking (van der Meulen, Van der Meulen et al., 2016). To unravel the neural responses in childhood, 7–9-year-old twins completed the same task as part of the L-CID study (van der Meulen et al., 2017, 2018). This study examined three processes in the PCG: (1) receiving tosses from the other players, which results in the personal experience of inclusion (2) not receiving tosses from the others, which results in the personal experience of exclusion, and (3) tossing to the excluded player, which results in the other-oriented behavior of non-costly helping. First, it was observed that experienced inclusion relative to not receiving the ball was associated with activation in the pre-SMA, whereas experienced exclusion was associated with activation in the anterior medial prefrontal cortex, subgenual ACC, and the inferior frontal gyrus (see Fig. 4). Second, prosocial helping compared to tossing to excluders was associated with activation in the precuneus, a region often implicated in self and other oriented processing (Pfeifer and Peake, 2012). Finally, the insula was also involved in prosocial helping but only in relation to individual differences in helping behavior. That is, participants who showed less prosocial helping recruited the bilateral insula more strongly when they tossed to the excluded player, possibly indicating that this is behavior that competes with their own dominant behavior and is therefore more salient (Rilling and Sanfey, 2011).

Taken together, adults recruit the ventral striatum and TPJ during prosocial behavior (van der Meulen et al., 2016), whereas in middle childhood prosocial helping is associated with increased activity in precuneus and performance-dependent insula (van der Meulen et al., 2018). A prior study that compared 12−17-year-old adolescents (n = 20) to young adults (n = 20) confirmed that in adults prosocial helping was associated with increased activity in the TPJ and superior medial prefrontal cortex, but this activity was stronger in adults than in adolescents (Tousignant et al., 2018). These results together suggest that processes that drive prosocial helping change over the course of development. Importantly, prosocial helping often occurs in context, for example when experiencing the risk of being excluded or when experiencing relief from not being excluded. In future studies it is therefore important to systematically vary these contextual factors for a full understanding of prosocial behaviors.

### 2.3 Relation between social evaluation sensitivity, aggression and prosocial behavior

**Fig. 5:**
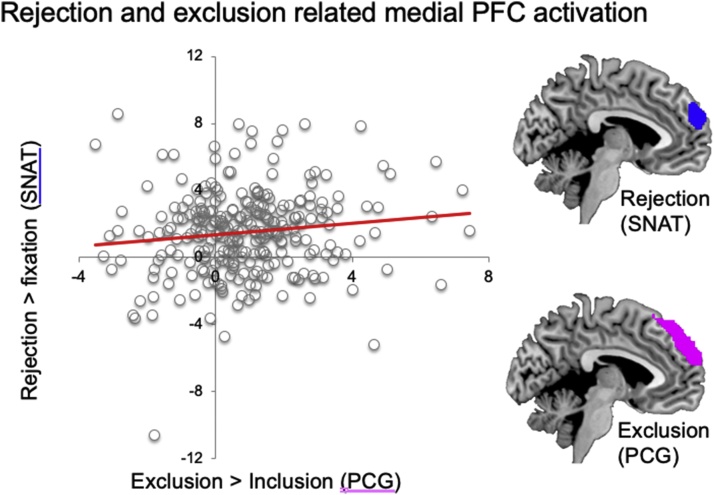
SNAT-rejection and PCG-exclusion related superior medial PFC activity from data reported in Achterberg et al. (2018a) and Van der Meulen et al. (2018) in 7-9-year old participants showing correlated activity across two paradigms in superior mPFC. Included data come from the contrasts: rejection-fixation (Achterberg et al., 2018a) and exclusion-inclusion (Van der Meulen et al., 2018), *r* = .17, based on a sample size of *n* = 261. Data were only included when the participants met the inclusion criteria for both experimental tasks.

The studies reported by Achterberg and colleagues (Achterberg et al., 2018a) and Van der Meulen and colleagues (van der Meulen et al., 2018) were conducted within the same L-CID sample, providing unique opportunities to examine the overlap in neural activity within the 7–9-year-old participants included in the study. Fig. 4 displays the neural overlap for receiving and responding to social feedback. First, social rejection (social negative feedback in the SNAT paradigm and experienced exclusion in the PCG paradigm) was associated with increased activity in superior medial prefrontal cortex in both paradigms, a region according to the Neurosynth analysis associated with social cognition. In contrast, receiving feedback that represented social acceptance (social positive feedback in the SNAT paradigm and experiencing social inclusion in the PCG paradigm) was associated with overlapping activity in SMA and DLPFC, regions that according to the Neurosynth analysis are associated with control processes. Note that in the SNAT paradigm participants were instructed to always press the noise button, but they were free to decide the length of the button press. DLFPC activity following positive feedback is therefore consistent with our predictions as this is the condition where participants put most effort into pressing the button as shortly as possible (Achterberg et al., 2020). Finally, receiving social feedback in general (positive + negative feedback relative to neutral feedback) in the SNAT paradigm was associated with increased activity in medial prefrontal cortex, a region according to the Neurosynth analysis associated with self-referential processing. These findings suggest that social evaluative feedback across social contexts is associated with activation in distinct regions of the medial prefrontal cortex. Interestingly, as can be seen in Fig. 5, neural activity in superior medial prefrontal cortex from the two task paradigms (SNAT and pCG) for rejection and experienced exclusion was correlated across tasks, using data previously published (Achterberg, van Duijvenvoorde, et al., 2018; van der Meulen et al., 2018). These findings highlight that there is some commonality in the neural response to negative social evaluation events that can be observed across experimental contexts. It is expected that self-protective responses also play a role in prosociality. Relatedly, positive social evaluation of self can trigger prosocial behavior. These intertwined relations between self-control and prosociality are consistent with the notion that self- and other-oriented processes are strongly aligned and rely on common neural regions (Crone and Fuligni, 2020). A question for future research is whether this commonality in neural responses is a stronger predictor for behavior and attitudes in comparison to single task measures.

### 2.4 A bi-dimensional taxonomy of social evaluation effects

An interesting approach for future research concerns the bi-dimensional influences of social evaluation sensitivity for self and others on developmental outcomes (Hawley, 1999). Self-protection mechanisms may harm social relations when one responds aggressively towards others, without concern for others in need. In contrast, aggressive mechanisms may be helpful when combined with prosocial tendencies in case others are harmed, because it enhances self-esteem (i.e., self-protection) while also responding to needs of others. Prior research has shown that such a bi-dimensional taxonomy is a useful direction to predict developmental outcomes (Sunami et al., 2019). This approach was previously applied to identify a specific group of adolescent youth who are prosocial risk-takers, thus, youth who score high on both rebellious behavior and prosocial tendencies (Blankenstein et al., 2019; Do et al., 2017). Research on adolescents also revealed that certain adolescents can be both aggressive and prosocial which can be a way to achieve social goals and status (Cillessen and Rose, 2005; Hartl et al., 2019). Individuals who use both strategies are considered to be the ones that are most responsive to their environment and adjust their behaviors to reach social goals (Hawley, 2002).

**Fig. 6:**
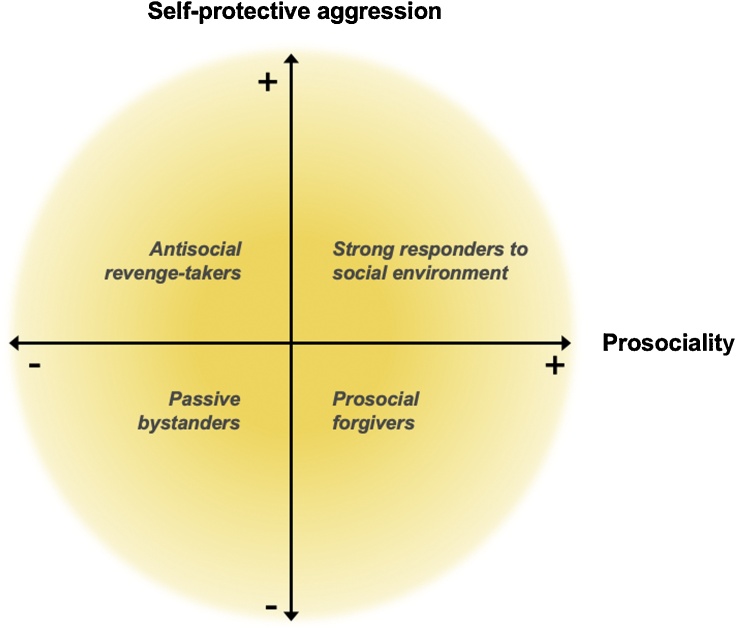
Behavioral profiles based on combined profiles of self-protective responses (aggression following personal rejection) and prosocial behavior (helping following observed rejection).

The overlap in neural activation in the medial prefrontal cortex suggests that this region is an important connecting region that responds to both negative social or rejection feedback of self and others. Behavioral responses to evaluations of self and other can be organized according to two axes: (i) the dimension of aggressive, self-protective acting-out tendencies: variance on this dimension may lead individuals to respond aggressively to others versus controlling their anger; and (ii) the dimension of prosocial tendencies: variance on this dimension may lead individuals to prosocially help others versus showing no signs of prosocial helping. This taxonomy leads to four groups (for ease of interpretation but continuous variation can be projected on the axes): The first group consists of individuals who show self-protective aggression combined with low levels of prosocial helping and who could be classified as anti-social revenge takers (Fig. 6A, left-hand upper quadrant). The second group consists of individuals who score high on aggression but also on helping others (right-hand upper quadrant). These individuals may be particularly aware of - and sensitive to - the environment. Individuals who control their aggression while also showing helping behavior towards excluded others can be characterized as prosocial forgivers (right-hand lower quadrant). Finally, the fourth group consists of individuals who show no aggression combined with low levels of prosocial helping; these are individuals who could be classified as passive bystanders (left-hand lower quadrant). In the latter group, there may be a subgroup that participates in exclusion, showing negative prosocial helping scores. Future research can determine whether these subtypes or combination of the two axes are predictive for longitudinal social and mental health outcomes. The combined perspective may lead to a more nuanced and specific analysis of neural and behavioral social competence indicators and predictors of social competence (Sunami et al., 2019).

## 3 Differential susceptibility to social experiences

The prolonged development of brain regions implicated in social evaluation suggests a window of sensitivity to environmental experiences. Regions from the social brain network, including the medial prefrontal cortex, TPJ and posterior STS show grey matter increases and decreases until the early twenties (Mills et al., 2016). We argue that periods of rapid growth may be associated not only with enhanced susceptibility to the environment, but also enhanced *differential* susceptibility on future outcomes. What are characteristics of youth who are particularly susceptible to better *and* worse (changes in) environments? Are there time windows in which differential susceptibility is enhanced? Can we identify neural markers of differential susceptibility?

### 3.1 Genetic influences

One approach to understand inter-individual differences in processes associated with social evaluation is examining the relative genetic influence on brain development and behavior (van Dongen et al., 2016). The classic within-twin phenotype comparison design allows for estimation of the proportions of genetic and environmental contributions to explaining the variance, by comparing the intra-pair correlations within twin pairs that are monozygotic and share 100 % of their genes, and twin pairs that are dizygotic and share on average 50 % of their genes. A higher correlation for monozygotic in comparison to dizygotic twins indicates a stronger influence of genetic factors. Correlations that are comparable for monozygotic twins and dizygotic twins indicates effects of shared environment. Variance that cannot be attributed to genetic or shared environment indicates either unique environment effects or measurement error. For further inspection, the structural equation ACE model (A: genetic influences, C: shared environmental influences, E: unique environmental influences) is the most commonly used approach to quantify the genetic and environmental contributions.

It has been shown that total grey matter volumes are highly heritable in adults (van Soelen et al., 2012). Heritability studies in infancy, childhood and adolescence indicate that there is evidence for genetic influences on brain growth, but in certain developmental periods estimates of the genetic influences are larger than in others (Polderman et al., 2015). For example, heritability of brain structures is already observed in infancy but is stronger for surface area (78 %) than for cortical thickness (29 %), and shows relatively little regional specificity (Jha et al., 2018), although a prior study suggested larger genetic influences on the posterior cortex compared to the prefrontal cortex in neonates (Gilmore et al., 2010). Moreover, heritability of white matter tracks was observed in twin studies including neonates, 1-year-olds, and 2-year-olds, but no evidence for heritability of white matter change between the age of 1 and 2 years (Lee et al., 2019). In older children and adolescents, heritability estimates are much higher (van Soelen et al., 2012) but genetic influence differs between regions in the brain, with more pronounced environmental influences in the social brain network than in visual and motor cortex (Van der Meulen et al., 2020). Understanding the genetic and/or environmental contributions and the changes in their impact across development may reveal potential windows of sensitivity to the environment during development.

The L-CID studies referred to in this review made use of the classic ACE twin design in two overlapping cohorts of 238 twin pairs (ages 3−9-years) and 256 twin pairs (ages 7−13-years). We found considerable heritability estimates for questionnaire trait phenotypes of prosociality and effort-full control (van Wijk et al., 2019a; Vrijhof et al., 2018), modest evidence for shared environmental and genetic factors explaining individual differences in (more state-like) behavioral aggression in the SNAT paradigm in 7–9-year-old children (Achterberg et al., 2018b) and 7−11-year old children (Achterberg et al., 2020), but limited evidence for genetic contribution to behavioral task performance on the PCG for 7–9-year-old children (van der Meulen et al., 2018). The differences in genetic versus environmental explanations of interindividual variation in prosociality, feelings of rejection and bias to display aggression might be related to the E component of the ACE modeling that represents unique environmental influences (making children within one family more different from each other) as well as measurement error. Parent- or self-reported attitudes or behaviors might be less valid but more reliable thus containing less measurement errors whereas pro- or antisocial behaviors measured in single test settings might be more indicative of state-like and context-dependent interactions increasing the E component.

In spite of the absence of behavioral evidence for genetic contributions, there was some evidence for genetic influences on neural signals in the range of 10–20 %, specifically for the DLPFC and supplementary motor area following positive feedback in the SNAT paradigm (Achterberg et al., 2018a), and the inferior frontal gyrus during experienced exclusion in the PCG paradigm (van der Meulen et al., 2018). Moreover, resting state connectivity analyses in the same participant sample revealed that in the 7–9-year-old children ventral striatum connectivity with ventromedial and orbitofrontal cortices was best explained by genetic influences with estimates between 23 % and 67 %. In contrast, subcortical-cortical connections between amygdala and ventral anterior cingulate cortex were best explained by shared environmental influences (Achterberg et al., 2018a). Together, these studies reveal that there is some evidence for genetic and shared environmental factors that impact neural signals during self/other evaluations and during resting state connectivity. However, future longitudinal studies are needed to understand stability versus change over time, and to test for sensitive time windows of (differential) environmental influences.

### 3.2 Social influences on social evaluation

Even though a real test of differential susceptibility requires measures of longitudinal within-person change (preferably in randomized control trials), some initial evidence for heightened susceptibility to the environment may already be obtained by studying how the variation in susceptibility of children and adolescents varies over time. We examined moment-to-moment task influences (so-called ‘nanotrials’, (Bakermans-Kranenburg and Van IJzendoorn, 2015)), as a means of testing how social experiences trigger different responses in children who are more or less susceptible because of prenatal or perinatal experiences or genetic make-up, and testing whether children differ most in their susceptibility during developmental phases of accelerated growth (Windhorst et al., 2015). Behavioral studies of social influences on adaptive behavior suggest that social influence by peers is more pronounced in early adolescence compared to adulthood, especially for the domains of risk perception (Knoll et al., 2015) and prosocial behavior (Foulkes et al., 2018). Recently, it was found that in adolescents compared to children and adults, social evaluation led to stronger rating adjustments of another peer who also had previously evaluated them (Rodman et al., 2017), and that children relative to adults adjusted their ratings based on immediate feedback rather than accumulated feedback (Yoon et al., 2018). These findings suggest that responses to negative social evaluation may change over the course of child and adolescent development, with larger effects of peers in early adolescence. These studies suggest that certain windows in development are associated with larger moment-to-moment sensitivity to environmental influences, but little is known about individual differences in sensitivity to these influences in terms of self-protection or resilience within these time windows.

### 3.3 Environmental influences on task performance and neural activity

Susceptibility to the environment can also be affected by previous supportive or aversive experiences as their stress-regulatory system might be pre-programmed to the environment they have to cope with at a later stage in their development (Ellis et al., 2011). For example, it was found that children who have a history of being socially excluded in the school context respond more strongly in terms of neural activity to the experience of social exclusion (Will et al., 2015a, 2015b), and to sharing goods between other participants and self, following social exclusion (Will et al., 2016). Girls who had experienced maltreatment in the family context also showed stronger neural activity to exclusion (van Harmelen et al., 2014). It is not yet well understood why some children are more resilient to such aversive experiences than others, which would require an in-depth evaluation of the interaction between potential susceptibility markers and social experiences (Van IJzendoorn et al, in press). The L-CID study incorporated a randomized control parenting intervention to test for sensitive windows to environmental change influences, and to test whether some children are more susceptible to such change than others. The Video-feedback Intervention to promote Positive Parenting and Sensitive Discipline (VIPP-SD) aims at enhancing sensitive interactions between parents and their children, and to support parents setting consistent but sensitive limit-setting when needed (Euser et al., 2016). By including two data waves preceding the intervention, and four data waves following the intervention, it will be possible to examine the effects of social enrichment on neural and behavioral development (Fig. 2). Furthermore, by including and combining two partially overlapping cohorts within an accelerated cohort-sequential design, we can study windows of increased sensitivity to the environment across ages 3–13 years (Fig. 1 and Fig. 2). Finally, we will use differential susceptibility markers to determine who benefits most from the intervention, that is, from a change for the better in the caregiving environment.

### 3.4 Neural differential susceptibility markers

Neural measures have previously been suggested as a possible new avenue for detection of early markers for (differential) susceptibility to the environment (Schriber and Guyer, 2015). One area that has been suggested as a promising candidate to be a marker of differential susceptibility is the ventral striatum (Do et al., in press). In prior research, this dopamine-rich reward-sensitive area was implicated in both negative developmental trajectories, such as risk taking and alcohol use (Braams et al., 2016), as well as positive developmental trajectories, such as prosocial helping and mental health (Telzer et al., 2014). Furthermore, dopamine levels have been indirectly implicated in differential susceptibility as dopamine-related genotypes were found to be markers of differential susceptibility in correlational and experimental studies (Bakermans-Kranenburg and Van IJzendoorn, 2015). Moreover, the ventral striatum shows high responsiveness in certain windows in development, specifically in mid adolescence (Braams et al., 2015; Silverman et al., 2015), which may indicate a sensitive window for differential outcomes. Finally, a study that examined the longitudinal development of fun seeking behavior, which is linked to ventral striatum activity (van Duijvenvoorde et al., 2014), showed that increases in fun seeking over time could explain trajectories of both rebellious and prosocial behavior (Blankenstein et al., 2019).

Here we propose that reactivity of the medial prefrontal cortex may be a similar differential susceptibility marker for sensitivity to social evaluation. The medial prefrontal cortex is strongly connected to both the ventral striatum and the lateral prefrontal cortex in terms of white matter connections (van den Bos et al., 2014), as well as functional connectivity (Achterberg et al., 2018a). The medial prefrontal cortex serves as a hub region for integrating self and other related neural processing (Crone and Fuligni, 2020). The medial prefrontal cortex shows stronger activity in adolescence compared to adulthood in social evaluation experiments (Blakemore and Mills, 2014), and it is characterized by a protracted developmental time course in terms of grey matter development (Mills et al., 2014). Finally, the medial prefrontal cortex is particularly sensitive to social evaluation in childhood, adolescence and adulthood (Achterberg et al., 2018b; Gunther Moor et al., 2010b; van der Meulen et al., 2018; Yoon et al., 2018). To be a marker of differential susceptibility, mPFC functioning should determine adaptation to the environment for better and for worse (Belsky et al., 2007; Belsky and Pluess, 2009). Indirect evidence for a potential differential susceptibility role of the prefrontal cortex comes from a study showing that adolescents with less cognitive control and associated neural activity showed lower social competence one year later when in a highly chaotic environment, relative to peers with higher cognitive control and neural activity. However, they did not show higher social competence when in a low chaos environment, so this result might be better explained in a double-risk model although a wider range of structured to chaotic environments might uncover a differential susceptibility cross-over interaction (Kim-Spoon et al., 2017). More direct evidence for differential susceptibility comes from a study in adolescent girls that demonstrated with cross-over interactions that the higher neural activity during social exclusion was associated with an increase in depression in girls with stressful parent-child relationships relative to girls with low neural reactivity, but lower levels of depression in girls with supportive parent-child relationships relative to girls with low neural reactivity (Rudolph et al., 2020). There is almost no research examining the neural development of the medial prefrontal cortex in early childhood, but our model would predict differential susceptibility in two important transition windows in development that are characterized by more rapid neuronal change: infancy/early childhood (Gilmore et al., 2018) and adolescence (Tamnes et al., 2017) (Fig. 1). An important question for future research will be to test whether neural sensitivity of the ventral striatum and the medial prefrontal cortex are early markers for differential susceptibility to environmental influences, which may impact developmental outcomes for better and for worse.

### 3.5 Multilevel markers of differential susceptibility

**Fig. 7:**
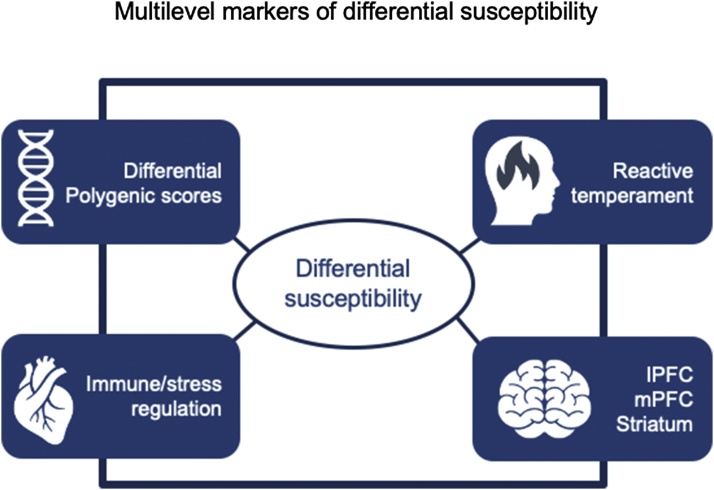
Four potential differential susceptibility markers on different levels of functioning. Differential polygenic scores are potentially the foundation for the three (endo-)phenotypical markers, but across development epigenetic changes in expression of the genes involved might result from environmental influences filtered through immune and neural reactivity or temperamental characteristics. childhood cohort (ECC, left) and middle childhood cohort (MCC, right).

Another challenging task for future research is to study the commonalities and differences between the various markers of differential susceptibility (see Fig. 7). First, going beyond single genes as markers of genetic differential susceptibility (Bakermans-Kranenburg and van IJzendoorn, 2006), polygenic susceptibility scores addressing specific developmental outcomes are promising candidates for genetic differential susceptibility, combining the moderating power of thousands of single nucleotide polymorphisms (Belsky and van IJzendoorn, 2017; Keers et al., 2016). Second, immune-system reactivity to stress has been among the first markers of differential susceptibility (Boyce, 2016; Boyce et al., 1995), which has been extended to stress regulation or reactivity more generally (biological sensitivity to context (Boyce and Ellis, 2005)). In a study on 5–6 year-old children, it was found that heightened cortisol response was associated with better executive functioning in children from higher socio-economic backgrounds and with lower performance in children from lower socio-economic backgrounds compared to their peers with lower cortisol response. Lower cortisol responses seemed to buffer the influence of socio-economic backgrounds (Obradovic et al., 2016). Third, ventral striatum reactivity has been suggested as a plausible marker of differential susceptibility (Do et al., in press; Schriber and Guyer, 2015). We hypothesize that the medial prefrontal cortex, and its connectivity to lateral prefrontal cortex and ventral striatum, provides a potential broader dynamic neural marker of susceptibility. Fourth, reactive temperament or an equivalent adult trait on the level of personality such as sensory sensitivity has been considered as a phenotypical marker of differential susceptibility (Belsky, 2005; Slagt et al., 2016).

In L-CID markers of differential susceptibility have been assessed at several longitudinal assessments. Furthermore, the longitudinal accelerated cohort-sequential design of the study covers an age range from early childhood to early adolescence. Lastly, the design includes a randomized control trial required for sufficient statistical power to test the moderating effects of the markers (van Ijzendoorn et al., 2011). The unique opportunity for future work in L-CID therefore is to examine the moderating role of each susceptibility marker in different developmental periods, to explore any relations among the markers, and test whether perhaps the commonality among various markers could be the most powerful aggregate marker of differential susceptibility (see Fig. 7). In Fig. 7 we do not suggest any causal influences between the different markers that are situated on different levels of neurobiological and behavioral functioning, from genetics to hormonal and neural functioning to temperamentally shaped behavior. The associations between the four markers are object of exploratory research as studies from which to derive specific hypotheses are still absent.

## 4 Conclusions

In this review, we present a neuroscientific differential susceptibility model that can be tested in future research and that is the driving motivation for the Leiden Consortium on Individual Development (L-CID) study. Several of the hypotheses can only be examined with longitudinal models including a randomized intervention, but the cross-sectional analyses provide important starting points for unraveling the dynamics of social evaluation. In future research, we will be able to extend these insights to contribute to answering the question: which children are most susceptible to environmental influences and at what time-points or windows in development are influences from the environment most pronounced, thus contributing to the perennial question in policy, prevention and therapeutic approaches: what works for whom at what time in development?

The L-CID study provides several new directions for understanding social evaluation in the context of self-protective mechanisms as well as other-oriented motivations. We proposed that the bi-directional relations between these processes will provide an important starting point for understanding social competence development and other developmental outcomes. Several important factors potentially explaining differences in social competence were not explicitly covered in this review, such as helping members of ingroup versus outgroup, and the influence of twin-sibling relationships. It is our ambition that in the future L-CID will provide a rich open data set for researchers to examine the processes involved in social competence and extend our work to new domains. By using a multi-method, multi-informant and multi-time-points approach, we hope to contribute to better operationalization of the core concepts, which ultimately can lead to better characterization of developmental problems and psychopathologies.

Although our study primarily aims at behaviors related to social competence, future studies may focus more strongly on strategic considerations or motivations for self-control and prosociality. This question has received most attention in the study of motivations for prosocial behavior (Thielmann et al., 2020). Paradigms examining costly versus non-costly helping, for example, suggest that different processes may drive these behaviors (Cutler and Campbell-Meiklejohn, 2019). Also, several findings show that prosocial behavior is dependent on the target and the presence of others (Foulkes et al., 2018; Guroglu et al., 2014), suggesting that prosocial behavior is strongly dependent on context (van IJzendoorn and Bakermans-Kranenburg, 2014). These findings may also explain why measures of prosocial behavior tend to diverge in experimental paradigms and questionnaire research, although indices of prosocial behavior are often combined across multiple contexts. Questionnaire research may be particularly sensitive to response bias, given that responding to questions about prosociality might entail self-protective and defensive mechanisms, but a strength of questionnaires is that multiple daily life contexts are covered. Relatedly, little is known about the context-specificity of self-control following social evaluation, for example, being rejected by a friend or caregiver versus an unknown other. Rejection by known versus unknown others may also differentially influence self-protection mechanisms, as rejection by a familiar other might cause feelings of depression whereas rejection by an unknown other might lead to acts of aggression. Future research should examine prosociality and self-control following social evaluation in terms of stability and change, and determine the contextual factors that influence behavior and neural activity, and brain-behavior associations.

In sum, we point towards activity and connectivity in the medial prefrontal cortex and the ventral striatum as potential neural differential susceptibility markers. In future research, these markers will be combined with insights from the intervention trial and other differential susceptibility markers (polygenetic scores, reactive temperament) to unravel why some children are more susceptible to environmental influences than others. An important aim will be to test stability and change of neural signals over time in order to contribute to a better understanding of reliability of measurement versus context-dependent change (Herting et al., 2017). Together with the Youth-CID program (this issue) and other large data sets including ABCD, Generation R, and other developmental studies around the world (Rosenberg et al., 2018), it will be possible to provide richer and culture- and context-dependent insights in neural and behavioral development during childhood and adolescence.

## Contributions

EAC and MHvIJ drafted the manuscript. MJBK contributed to the study design and commented on the first draft of this manuscript. All other authors contributed to the design, data collection, analysis and final manuscript and are listed in alphabetical order.
