# Supplementary material for: Neural and behavioral signatures of social evaluation and adaptation in childhood and adolescence: The Leiden consortium on individual development (L-CID)
Source: Dev Cogn Neurosci. 2020 Jul 11;45:100805. doi: 10.1016/j.dcn.2020.100805 (PMC7390777; doi:10.1016/j.dcn.2020.100805)
Supplement: Supplementary file 3 [file mmc3.docx]

|  | **Early Childhood Cohort** | **Middle Childhood Cohort** |
| --- | --- | --- |
| N | 476 | 512 |
| Girls | 51% | 51% |
| Right handed | 86% | 87% |
| Caucasian | 96% | 91 % |
| Monozygotic | 57% | 55 % |
| AXIS-I disorder^1^ | 0 | 11 (2.1%)^1^ |
| Primary parent female | 92% | 91 % |
| SES low - middle - high^2^ | 6% - 37% - 55% | 9% - 46% - 45% |
| Age (SD) | 3.86 (0.58) | 7.94 (0.67) |
| Age range | 2.86-5.48 | 7.02-9.68 |
| Mean IQ (SD) | 102.89 (10.75) | 103.58 (11.76) |
| IQ range | 65-130 | 72.50-137.50 |
| *^1^ 9 ADHD/ADD; 1 PDD-NOS; 1 Generalized Anxiety Disorder*  *^2^ Social Economic Status, based on parental education* | | |

**Table A3** Demographics of the early childhood cohort (ECC) and middle childhood cohort (MCC).


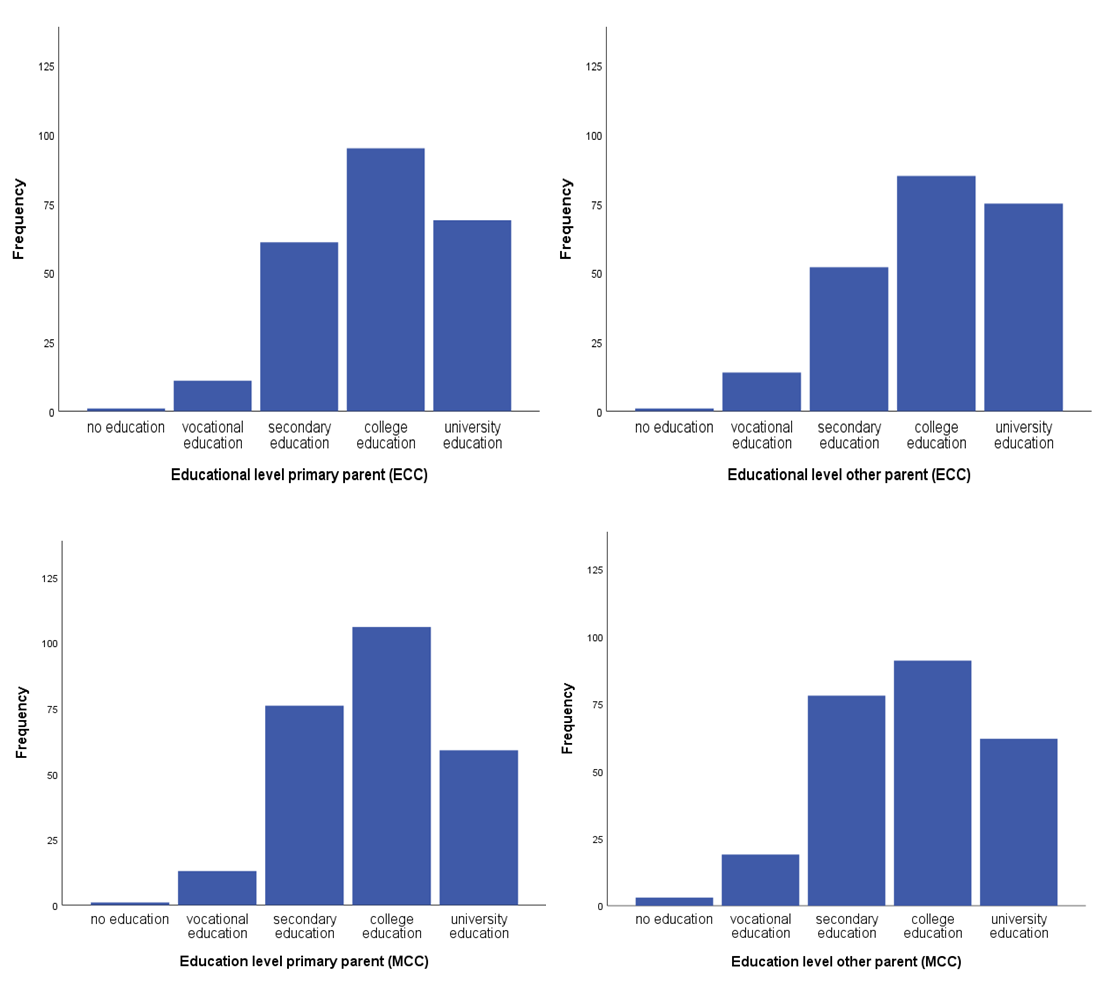


**Figure A3.1** Parental education level for the primary parent (left panel) and the other parent (right panel) in the early childhood cohort (ECC) and middle childhood cohort (MCC).

**
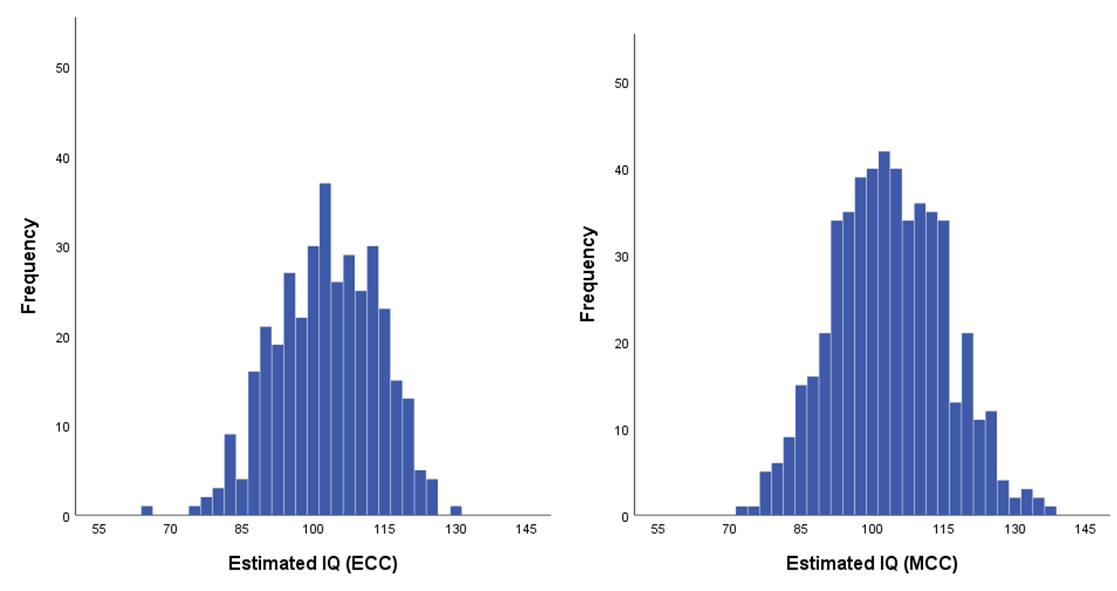
**

**Figure A3.2** Distribution of estimated inteligence quofficient (IC) for the early childhood cohort (ECC, left) and middle childhood cohort (MCC, right).
